# Supplementary material for: Inferring a network from dynamical signals at its nodes
Source: PLoS Comput Biol. 2020 Nov 30;16(11):e1008435. doi: 10.1371/journal.pcbi.1008435 (PMC7728228; doi:10.1371/journal.pcbi.1008435)
Supplement: Attachment — Submitted filename: Response_to_Reviewers_PLoS.pdf [file pcbi.1008435.s001.pdf]

LAUFER CENTER
FOR PHYSICAL AND QUANTITATIVE BIOLOGY

September 8, 2020

Dear Editor,

We believe we have fully addressed the comments of the reviewers. Most importantly, we have better explained why and when this is the method of choice, not just Max Cal, but this specific approximation of it. We give more intuition on why it works in our examples. Below are the detailed responses. Thank you.

Sincerely,

Ken A. Dill

Louis & Beatrice Laufer Endowed Chair in Physical Biology
Distinguished Professor of Chemistry & Physics
Director, Laufer Center for Physical and Quantitative Biology
Stony Brook University
Stony Brook, NY 11794-5252
(631) 632-5400

# Reviewer 1:

*Weistuch et al provide an approximate solution to inferring network structure given temporal signal strength using Maximum Caliber. The authors show that this approach can infer the appropriate network constraints of the classic Collins Toggle switch, and a more complex brain neural network. Overall, the idea is interesting and the results are compelling. However, the use of network inference, such as Bayesian-type and ML inference models for inferring optimal network topologies is not a new notion on its own, and the authors did not sufficiently cast their results in the context of the (very widely established) field. Therefore, it was unclear how, and under what conditions, this approach performs comparably/superior etc. The authors should both elaborate with text, and use simulations, to contrast the advantages and/or utility of their approach to other established ones; this will better elucidate the overall constraints on performance that were not sufficiently described or explored. Moreover, given the abundance of available temporal scientific data and known network topologies, the authors should demonstrate the accuracy of this method using actual experimental data, rather than entirely simulated experiments. Without that, it's difficult to assess the capabilities and limitations of the author's approach compared to other's that have been tested and previously implemented. Moreover, this will elucidate the range of experimental variability that it can sustain, which currently is unclear in the manuscript.*

While we thank the reviewer for these insightful comments, we feel that the reviewer may have misinterpreted the purpose of our manuscript and the methodological advance that we give here. This reviewer sees our method as a better tool for inferring the connectivity between a network of nodes. However, the real advantage here is the ability to infer not just connections, but trajectory distributions on networks. And, unlike other approaches, our method does not require prior knowledge or assumption of the underlying structure of the system we are trying to infer. We have added clarifications.

So, the examples we chose are synthetic, having a known ground-truth, because that's the relevant proof we need here. In addition, our examples are chosen for the new insights of the method into previously elusive biophysical questions. In particular, we demonstrate that our Uncoupled Max Cal method can identify (analytically) the critical point of the genetic toggle switch, which had previously been only attainable numerically (See Pressé J. Phys. Chem. B 2011). Similarly, our neural network model was designed according to previously described parameters (See Mora PRL 2015 and Schneidmann Nature 2006). The challenge here is that inferring the dynamic relationships between a general population of neurons has been a computationally challenging problem.

We have included a new Method section entitled "Quantifying the accuracy of Linear Coupling". This section tells what conditions are sufficient for the accuracy of our model and the upper bounds on the typical error one might expect.

# Reviewer 2:

## Major Points:

1. *Overall, I think providing an intuition for why each of the two approximation schemes work would be helpful for someone like me. For example, in the case of uncoupled Max Cal approximation, I was surprised to see that one can still infer the connectivity between the two nodes in a flip-flop switch despite assuming K_{12} = K_{21} = 0. Clearly, the math works and matches the exact simulation results in Fig. 2. But I still don't understand intuitively or at a deep level why this works.*

   Using the toggle switch as an example, Uncoupled Max Cal works because typical fluctuations in protein numbers have a minimal effect on the repression of the opposing gene. When the effect of these fluctuations is larger, we need a higher-order (such as Linear Coupling) approximation. We have now added a more thorough discussion of the intuition behind our methodologies in a new section entitled " When to use the different approximations".

2. *(Related to A) Add a section that guides the experimentalist on when one can use each approximation scheme. Since we do not, a priori, know the network structure in the inverse problem, we cannot check whether the approximated h_i and K_{ij} are "correct" or not by other methods (i.e., unlike in the two examples - Fig. 2 and Fig. 4 - we don't have a "Ground Truth" model in many situations). So I was hoping to see when I can use either one of these two approximations when I'm faced with an unknown network. Any insights on this would be helpful for practical purposes (either in words or some inequalities that one should satisfy for using each approximation).*

   We have now addressed this in our new section entitled "When to use the different approximations". In addition, as noted in response to R1 above, our new Method section "Quantifying the accuracy of Linear Coupling" gives the typical errors to expect for each method.

3. *In the toggle-switch example, it seems to me that, to use the method, one needs more than just the integer number of the two gene-products: N_A(t) and N_B(t) The method also uses l_alpha and l_beta - the bursts of proteins A and B produced within interval dt (Appendix C uses N_A, N_B, l_alpha, l_beta). This is different from the simpler description given in "The Problem" section which mentions one time series: v(t) = {v_1(t), ..., v_N(t)}. Is there a general recipe for what constitutes a relevant time series for a given network? Why isn't just {N_A(t), N_B(t)} sufficient as a time-series and how can we determine, in other networks, what is sufficient as a time-series data? I think that a new section which gives a practical guidance on this in a new section will make the work more useable by experimentalists.*

   In general, any new added constraint requires another measured quantity. For the toggle switch example, these constraints have previously been specified as the average production, degradation, and correlation between the different protein species A and B (see Pressé J. Chem Phys. B 2011). The constraints come from the problem, not the method. For the general question of how to choose the constraints in Max Cal, we refer to the previous literature (Pressé RMP 2013, Dixit JCP, 2017, and Ann. RPC Ghosh 2020).

4. *Related to (A-C): A section that describes a step-by-step procedure (like an instruction manual) that an experimentalist can use for implementing the approximation schemes would be helpful.*

   As noted for R1, we added a section "When to use the different approximations". For the step-by-step details, we refer to previous literature on prior application of Max Cal to genetic circuits (See Pressé J. Phys. Chem. B 2011 and Firman J. Phys. Chem. B. 2018).

5. *In the toggle-switch example, does the uncoupled Max Cal approach fail only at the critical point (K_c)? What about sufficiently close to the critical point? A figure that shows the deviation between the exact and approximation as K approaches K_c would be nice.*

   We did not add a new figure here, because our current Figure 4d answers essentially that question.

Stony Brook University, NY 11794-5252 • Phone: 631.632.5400 • Fax: 631.632.5405

6. *To use the method, an experimentalist must measure a time-series for a certain time interval (0, T). How should one choose the T, especially if the connectivity varies drastically over time? Take data for (0, T) and for (0, 2T) and see if there's convergence in the values? Some guidance on this in the conclusion or a new section would help experimentalists.*

   We discuss in the main text that the time T is the length (in arbitrary units) of the data stream provided to the modeler. This is always a pre-processing choice before Max Cal (or any modeling for that matter) is applied.

7. *Direct versus indirect connections between nodes. I wasn't sure how one would distinguish node A being directly connected to B (e.g., in flip-flop switch) versus A being indirectly connected to B through an intermediate node C (e.g., feed-forward pathway). Some comments on this towards the end would be helpful.*

   We have added a sentence; Max Cal infers only direct interactions between nodes.

8. *I would lengthen the Conclusion section to summarize some existing methods for inferring network structure and how the authors' method goes beyond those. For example, I think the authors can cite Kholodenko et al. PNAS (2002): https://www.pnas.org/content/99/20/12841 --- like this PNAS paper, most methods that I and others know of for inferring a network structure use static data and, most importantly, require perturbing each potential connection between two nodes and then measuring the resulting response (a snapshot in time with, for example, RNA FISH). The latter is impractical for large networks and even for smaller networks, due to lack of small molecules for specific perturbations and/or infeasible numbers of mutants to make. The current manuscript distinguishes itself by not requiring such perturbations (just require endogenous network) and a time series rather than static snapshots.*

   As the reviewer keenly notes, our method infers the coupled dynamics between nodes in a network without requiring any additional experiments. We have now added text noting these advances.

# Minor Points:

1. *Suggestions on higher order (2nd order and above) perturbations mentioned at the end of Pg. 4. -- Clarify whether these are speculations or they are sure to work.*

   These perturbations are sure to work (see Tersenghi J. Stat. Mech. 2012). We have made a minor change in this sentence to clarify this point.

2. *Fig. 2b: Label the y-axis in Fig. 2b. These are Master-equation simulations (Gillespie algorithm)? Specify in the caption.*

   The label (N) has now been added to the y-axis in Fig. 2b. The simulations were performed using the trajectory distribution given by Max Cal (Eq. 24, with known Lagrange multipliers). A comparison of Max Cal to the master equation (simulated using the Gillespie algorithm) has already been done in a previous work (See Pressé J. Phys. Chem. B 2011). This has now been stated in the caption of Fig. 2b.

3. *Fig. 2d: label y-axis as P(N).*

   Done.

4. *Appendix C states that N_A and N_B are numbers at the beginning of each dt-interval. How is this consistent with the N_A(t) and N_B(t) in Fig. 2 and the main text?*

   dt can be arbitrarily small (depending on the resolution of the data). We don't see any issue here.

5. *Description of toggle switch: I think it's more correct to say that N_A and N_B are mRNA levels measured in live cells with techniques like MS2 in E. coli (c.f. Ido Golding's work) rather than proteins; I'm not aware of ways to measure integer numbers of proteins in live or dead cells.*

   In the associated references (See Pressé J. Phys. Chem. B 2011 and Firman J. Phys. Chem. B. 2018), protein numbers are inferred using fluorescence data. We have now specified this.

# Reviewer 3:

1. *Since the audience might not be familiar with the insight and strength of Max Cal, they would benefit from a concise review of the status of the field, past applications of Max Cal, why it is the method of choice for inferring dynamic processes, and what are the potential limitations, in the Introduction. This would help motivate the development of approximate approaches and establish the novelty of this work.*

   We have now added a few sentences describing the prior theoretical developments and biological applications of Max Cal to emphasize that Max Cal is a general and proven method for inferring dynamics in noisy biological systems given only limited experimental information.

2. *To demonstrate the value of this new development, it would be very helpful to show how the performance of approximate Max Cal compares to alternative approaches for inferring interactions in a network. Such comparison would help the reader to appreciate why and when this approximate approach is a good choice.*

   The key difference between Max Cal and other approaches for inferring interactions in a network is that Max Cal seeks to infer a probability distribution for how these networks evolve over time without being tied down to specific model assumptions. Thus, Max Cal seeks to answer a different set of questions from other commonly used tools for inferring networks. We have now added a few sentences (and references) in the introduction to further illustrate this point.

3. *Can the authors quantify in some way the regime of applicability of the approximate solutions? In other words, under what conditions (e.g. general features of the network) shall we expect the approximations to be quantitatively useful?*

   We have now added a new Method section entitled: "Quantifying the accuracy of Linear Coupling: to illustrate the wide regime of applicability of our approaches. In addition, we have added text (in particular, a new section entitled "When to use the different

approximations") describing the specific conditions where each method should be used and why. For further details, we refer to our responses to reviewer 2.

4. *Since this work sets out to address the challenge of high dimensionality of the inference problem, it would be more convincing if the result can be demonstrated for a larger system, e.g. a genetic regulatory network with coupled modules. How would the approximate solutions perform in cases of more complex nonlinearities and feedback than those in a single toggle switch?*

While we thank the reviewer for this insight, we felt that adding additional complexity to our examples would obscure the insights provided by our methods. In addition, our examples were chosen in order to compare to the challenges faced in previous works. Finally, based on previous literature on mean-field theory, we have no reason to doubt that our method is fully general.

5. *Can the authors comment on the broader insights gained from these two particular examples? E.g. why can the uncoupled solution capture the critical point and full distributions away from it?*

Mean-field approaches are often used to identify steady-states (or free energy minima) in complex, high-dimensional systems. Uncoupled Max Cal allows us to infer the dynamics of these complex systems when the interactions between our agents (but crucially not the agents themselves) can be approximated by their steady-state values. This approximation breaks down near critical points, when the effects of fluctuations can no longer be neglected. As is the typical approach in mean-field theory, these points can be identified by interpolating our solutions from both sides of the critical point.

When we are interested in the dynamics of these transient fluctuations (such as the avalanches that we described in our neural example), Uncoupled Max Cal is no longer sufficient. The insight provided by our second example, however, is that Linear Coupling Max Cal indeed correctly describes the properties of these fluctuations even close to critical points.

We have now added further clarification for these points by adding the additional section entitled "When to use the different approximations".

6. *To obtain the dynamic mean field equations in the linear coupling approximation, reverse KL divergence between the true Max Cal distribution and the approximate one is minimized. Can the authors clarify why "the greatest insight comes from choosing the former" (i.e. minimizing with respect to the constraints while keeping the Lagrangian multipliers fixed)? Also, is there an intuitive way to see that, to the leading order, solutions from minimizing the forward and reverse KL divergence would match?*

   We have rewritten this sentence to clarify that our choice was the most direct way to arrive at the correct solution. The solution obtained by minimizing the forward KL divergence suggests that we preserve the means and autocorrelations of each agent. However, in so doing, the external fields, h, will change. The reverse KL solution provides a way to correct for these changes by intuitively subtracting off the contribution to this field due to the interactions with other agents.

   We have also added an additional sentence to our section "How to choose the Uncoupled distribution" to relay this intuition to the reader.

7. *A minor point: in SI equations B2 and B3, should the first term be entropy (S instead of C)?*

   To avoid this subtle point, we have now explicitly written this term out as -<logp>.

100 Nicolls Rd, Stony Brook, NY 11794, 631-632-5400
Stony Brook University
